# Another piece in the progranulin puzzle: special binding between progranulin and prosaposin creates additional lysosomal access: An Editorial Comment for ‘The interaction between progranulin and prosaposin is mediated by granulins and the linker region between saposin B and C’ on page 236

**DOI:** 10.1111/jnc.14125

**Published:** 2017-08-04

**Authors:** Philip Van Damme

**Affiliations:** ^1^ Department of Neurosciences Experimental Neurology and Leuven Institute for Neuroscience and Disease (LIND) KU Leuven – University of Leuven Leuven Belgium; ^2^ Laboratory of Neurobiology VIB Center for Brain & Disease Research Leuven Belgium; ^3^ Department of Neurology University Hospitals Leuven Leuven Belgium

## Abstract

Loss‐of‐function mutations in the gene encoding the growth factor progranulin cause degeneration of the ageing brain in a dose‐dependent manner. While heterozygous mutations result in adult onset frontotemporal dementia, the much rarer homozygous null mutations cause an early onset lysosomal storage disorder. A better understanding of the biology of progranulin in the central nervous system is needed to find solutions for these incurable diseases. This Editorial highlights a study by Zhou *et al*. in the current issue of the *Journal of Neurochemistry*, in which the authors provide data that are a step towards this goal. Progranulin is mainly expressed by neurons and microglia and, although it is a secreted protein, it also ends up in lysosomes. Recently, the trafficking of progranulin and the molecular players involved have become better understood. A special interaction between progranulin and its travelling companion, prosaposin, explains how both proteins can use each other's transport receptors to gain access to lysosomes.

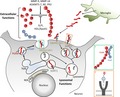

**Abbreviations used**
ADAMTS‐7: a disintegrin and metalloproteinase with thrombospondin motifs 7
HDL/ApoA1: high density lipoprotein/apolipoprotein A1
LRP1: low density lipoprotein receptor‐related protein 1
M6PR: mannose 6 phosphate receptor
MMP: matrix metalloproteinase
NE: neutrophil elastase
PGRN: progranulin
PR3: proteinase 3
PSAP: prosaposin
RER: rough endoplasmatic reticulum
SLPI: secreted leukocyte protease inhibitor
SORT1: sortilin
TGN: trans Golgi network

Progranulin is a pleiotropic growth factor involved in many processes including cell division, cell survival, inflammation, wound healing, and tumor growth. Human genetic studies have first identified the significance of progranulin in the aging brain, on one hand, and in lysosomes, on the other. In 2006, the important role of progranulin in the aging brain became clear with the discovery that heterozygous loss‐of‐function mutations in the progranulin gene (*GRN*) cause familial frontotemporal dementia (FTD) (Baker *et al*. 2006; Cruts *et al*. 2006). FTD is a dementia syndrome, characterized by neurodegeneration in the frontal and anterior temporal lobes. It affects people in mid age and, together with Alzheimer's disease, it is the most common form of dementia under 65. Among the different neuropathological subtypes, FTD caused by *GRN* mutations is characterized by neuronal inclusions containing the protein TDP‐43 (Mackenzie and Neumann 2016). The pathogenic mutations in *GRN* result in reduced progranulin protein levels, which make haploinsufficiency the underlying disease mechanism. In 2012, the importance of progranulin in lysosomes became clear as homozygous loss‐of‐function *GRN* mutations were shown to cause the lysosomal storage disorder, neuronal ceroid lipofuscinosis (Smith *et al*. 2012). Moreover, progranulin knock‐out mice display features reminiscent of neuronal ceroid lipofuscinosis (Gotzl *et al*. 2014). This added the *GRN* gene to a growing list of genes of which rare homozygous loss‐of‐function mutations are associated with an early‐onset lysosomal storage disorder, while the more common heterozygous mutations entail the risk of developing an age‐related neurodegenerative disease.

The impact of a progranulin shortage on the nervous system is the subject of intensive research. Progranulin is mainly expressed in neurons and in activated microglia. Multiple roles have been attributed to progranulin in the brain: neurite outgrowth, neuronal survival, microglial activation, neuroinflammation, phagocytosis of inflammatory cells, neuronal connectivity, and lysosomal function (Petkau and Leavitt 2014). Progranulin is a heavily glycosylated secreted protein, which nevertheless also ends up in lysosomes. It thus seems to carry out both extracellular and lysosomal functions.

Recent research has uncovered some of the mechanisms of the subcellular distribution of progranulin and the molecular players involved in this process (Fig. 1). Progranulin contains seven and a half granulin domains. Each consists of cysteine‐rich motifs that form disulfide bonds, responsible for a compact structure of the granulin domains. Progranulin also contains a signal peptide which directs it to secretory vesicles. After secretion, progranulin can be decomposed into individual granulins after cleavage at the linker regions between the granulin domains. Both the precursor protein and the individual granulins can be biologically active. The sorting receptor sortilin (SORT1) was identified as an important plasma membrane receptor for progranulin, which mediates endocytosis and the fast delivery of progranulin to lysosomes (Hu *et al*. 2010). Most likely, SORT1 is also able to carry progranulin directly from the Golgi apparatus to the lysosomes. Progranulin is coupled to SORT1 via the three last amino acids at its C‐terminus, but this binding seems not to be required for its neurotrophic functions. SORT1‐mediated endocytosis of progranulin does regulate the extracellular levels of progranulin, as reduced SORT1 levels cause an accumulation of extracellular progranulin (Hu *et al*. 2010).

With regard to the subcellular trafficking of progranulin, important lessons have also been learned by analogy with prosaposin, another heavily glycosylated protein which shares many structural and functional similarities with progranulin (Zhou *et al*. 2015). Prosaposin is a precursor protein that can be cleaved into four saposins (saposin A, B, C, and D). These saposins function as lysosomal sphingolipid activator proteins that help in the hydrolysis of sphingolipids by specific lysosomal hydrolases. Prosaposin is also secreted and can exert neurotrophic functions, but the main function of its individual saposins is confined to the lysosomal compartment. Saposins also adopt a compact structure as a result of disulfide bonds formed between cysteine residues. The compact structure and glycosylation of progranulin and saposins are thought to be important in resisting the lysosomal proteases as well as a low pH. Prosaposin also relies on trafficking receptors for its delivery to the lysosome using both the mannose‐6‐phosphate receptor and the low density lipoprotein receptor‐related protein 1 (LRP1). Prosaposin can travel directly from the Golgi network to lysosomes or can be taken up from the extracellular space by receptor‐mediated endocytosis and delivered through the endosomal pathway to the lysosomes. Although the roles of saposins in the lysosome are fairly well‐known, the lysosomal functions of progranulin have received less attention. Two recent studies suggest that progranulin functions as a lysosomal chaperone for β‐glucocerebrosidase and cathepsin D, two other proteins of which a deficiency is also associated with a lysosomal storage disorder (Jian *et al*. 2016; Beel *et al*. 2017). Interestingly, homozygous loss‐of‐function mutations of *prosaposin* or individual saposin domains also cause different types of an early onset lysosomal storage disorder. It is tempting to speculate that heterozygous loss‐of‐function *cathespin D* mutations could be linked to age‐related neurodegenerative disorders as well.

**Figure 1:**
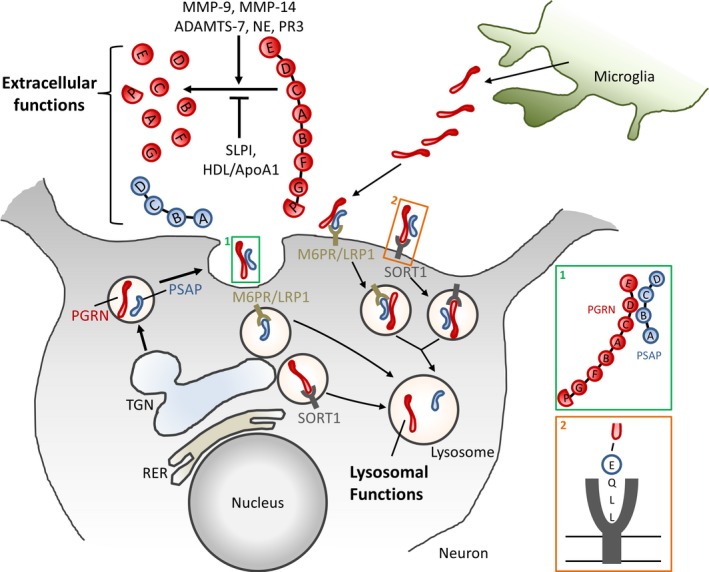
Schematic representation of progranulin and prosaposin transport. Progranulin and prosaposin are secreted proteins. Direct interaction between progranulin and SORT1, and between prosaposin and LRP1 or M6PR results in endocytosis and lysosomal delivery of the ligands. Because both ligands interact, they can indirectly use each other's transport system.

In 2015, Zhou *et al*. (2015) first showed that progranulin and prosaposin are not only merely analogous proteins, but that they actually pair up, helping each other to get transported to the lysosome. Prosaposin could facilitate a transport route to the lysosome for progranulin using M6RP or LRP1, independent of SORT1. More recently, the same group demonstrated that progranulin could do the same job for prosaposin, by adapting prosaposin to use a SORT1‐mediated lysosomal delivery (Zhou *et al*. 2017b). This leads to the concept that progranulin and prosaposin are traveling companions that can help each other's transport. Yet, the exact nature of this collaboration remained unclear. In a follow‐up study published in this issue of the journal, the interaction between progranulin and prosaposin is further clarified, which explains how progranulin and prosaposin facilitate lysosomal trafficking by smuggling each other through specific trafficking receptor‐mediated transport systems. In a beautiful series of immunoprecipitation experiments using different progranulin truncation constructs, the authors could show a direct interaction between different granulin domains and the linker region between saponin B and C (Zhou *et al*. 2017a). Especially the two most C‐terminal granulin domains, granulin D and E, had a high affinity to bind this linker region of prosaposin. This specific interaction between progranulin and prosaposin enabled progranulin to sneak in through the mannose‐6‐phosphate receptor or LRP1 mediated transport machinery. Many of these experiments were performed in fibroblasts, cell lines, and primary cortical cultures and therefore, the *in vivo* relevance remains to be proven. However, the redundancy in traveling routes for progranulin and prosaposin suggests that a safety margin is built in to secure the safe delivery of both proteins to the lysosomes, which underscores the importance of both proteins in the lysosome. Yet, several questions remain: If prosaposin can efficiently increase the delivery of progranulin to the lysosomes, why are heterozygous loss‐of‐function mutations in the *GRN* gene then not better tolerated? What proportion of progranulin and prosaposin in the lysosomes is taken up from the extracellular space? To what extent is lysosomal dysfunction responsible for the neurodegenerative phenotype in patients with FTD? And, finally, is the lysosomal dysfunction responsible for the reduced clearing of TDP‐43, thus causing the TDP‐43 accumulation and aggregation? A better knowledge of the functions of progranulin in the nervous system, within different cell types and subcellular compartments, will help to guide progranulin supplementation strategies.
